# Supplementary material for: Complexome profiling of the Chlamydomonas psb28 mutant reveals TEF5 as an early PSII assembly factor
Source: Plant Cell. 2025 Mar 17;37(6):koaf055. doi: 10.1093/plcell/koaf055 (PMC12164590; doi:10.1093/plcell/koaf055)
Supplement: koaf055_Supplementary_Data [file koaf055_supplementary_data.zip › psb28_interactive.zip!/plots/intensity/qualityPlots/peptides/AST1_Cre09.g387726.t1.1.html]

### Cre09.g387726.t1.1

Trivial name: AST1  
SeqCoverage [median]: 10.60  
Mass: 46.53 kDa  
  
 

| Sequence | MissedCleavage | Mass [Da] | wtIntensity | muIntensity | Score |
| --- | --- | --- | --- | --- | --- |
| ELALGPSHPAIR | 0 | 1259.70 | 1019.70 | 0.00 | 61.35 |
| TEEGKPYVLR | 1 | 1190.63 | 1903.41 | 1896.43 | 60.52 |
| VATVQALSGTGALR | 0 | 1342.76 | 1402.25 | 5078.57 | 142.08 |
|  |  |  | 4325.4 | 6975.0 |  |
